# Label accuracy of unregulated cannabidiol (CBD) products: measured concentration vs. label claim

**DOI:** 10.1186/s42238-022-00140-1

**Published:** 2022-06-06

**Authors:** Erin Johnson, Michael Kilgore, Shanna Babalonis

**Affiliations:** 1 Department of Pharmacology and Nutritional Science, University of Kentucky College of Medicine, Lexington, USA; 2 Department of Behavioral Science and Center On Drug and Alcohol Research, University of Kentucky College of Medicine, Lexington, USA

**Keywords:** Cannabidiol, CBD, Label accuracy, Label claim, Regulatory, Epidiolex

## Abstract

**Background:**

The legalization of hemp in the USA has led to tremendous growth in the availability of hemp-derived products, particularly cannabidiol (CBD) products. The lack of regulatory oversight in this industry has resulted in the marketing and sale of CBD products with questionable ingredients and quality. The aim of the current study was to examine the CBD content in 80 commercially available hemp-derived CBD products purchased from online and local retailers. Epidiolex® was also included in the study as a positive control.

**Methods:**

Hemp-derived CBD products were selected to represent products readily available to residents of Central Kentucky. The samples were comprised of local and national brands produced in a variety of locations inside and outside of Kentucky. The products were analyzed by liquid chromatography-tandem mass spectrometry (LC–MS/MS), and the analytical findings were compared to the label claims for CBD content. Descriptive statistics and normal-based confidence intervals were calculated using Microsoft Excel.

**Results:**

The label claims for CBD content ranged from 7.5 to 60 mg/mL, while LC–MS/MS analysis detected a range of 2.9 to 61.3 mg/mL. Of the 80 products evaluated, 37 contained CBD concentrations that were at least ± 10% different than the concentration listed on the label (range of 0.9 to 30.6 mg/mL from label claim) — 12 products contained < 90%, while 25 products contained > 110%. The degree of concordance for the samples tested using ± 10% tolerance from label claim was 54%.

**Conclusions:**

These data suggest that additional regulation is required to ensure label accuracy as nearly half of the products in this study were not properly labelled (i.e., not within a ± 10% margin of error). Consumers and practitioners should remain cautious of unregulated and often-mislabeled CBD products due to the risks of taking too much CBD (e.g., drug-drug interactions, liver enzyme elevations, increased side effects) and the consequences of taking too little (e.g., no clinical benefits due to underdosing). The results of this study support the continued need for good manufacturing practices and testing standards for CBD products.

## Background

Cannabidiol (CBD) is a nonintoxicating component of *Cannabis sativa* that has been the subject of increasing interest due to its purported therapeutic benefits. For decades, the close association of CBD to Δ^9^-tetrahydrocannabinol (Δ^9^-THC) (and previous schedule 1 status of CBD) hampered the research of the potential medicinal benefits. The landscape has been changing since, 2018 (1)the passage of the Agricultural Improvement Act of 2018 (2018 Farm Bill) which legalized products derived from hemp, defined as the plant/plant parts of *Cannabis sativa *L. with a Δ^9^-THC concentration of no more than 0.3% of dry weight, and (Agricultural Improvement Act, 2018) and (2) the removal of CBD from the US Drug Enforcement Administration (DEA) list of controlled substances. This legalization of hemp and hemp-derived products has led to rapid growth in the CBD industry. Epidolex®, a purified oral solution of CBD, was approved by the US Food and Drug Administration (FDA) in June 2018 and is now approved for the treatment of three forms of epilepsy, Lennox-Gastaut, Dravet syndrome, and epilepsy associated with tuberous sclerosis (Corroon and Kight 2018; FDA approves new indication for drug containing an active ingredient derived from cannabis to treat seizures in rare genetic disease [press release] 2020). With the exception of Epidiolex®, CBD products are largely unregulated in the USA and currently considered neither drugs nor legal dietary supplements (Gurley et al. 2020). Although the FDA has utilized the authority under the Federal Food, Drug, and Cosmetic Act (FD&C Act) to enforce some regulation of CBD products (e.g., false marketing claims) (Wagoner et al. 2021), there has been public pressure for the FDA to establish clear regulatory guidelines for CBD products.

Specifically, the FDA held a public hearing in June 2019 regarding CBD regulation and heard concerns from scientists regarding the chemical constituents of unregulated CBD products, including contamination from fungus, harmful by-products from the manufacturing process, and the presence of dangerous drugs (JWH compounds, cathinones) (FDA May 31, 2019) (Scientific data and information about products containing cannabis or cannabis-derived compounds 2019). The FDA and several research groups have also examined CBD concentrations in products and have reported generally consistent findings indicating concern with the label accuracy. Between 2015 and 2016, the FDA issued warning letters to 14 businesses with products containing less CBD than indicated on the label including instances of CBD content being negligible or less than 1% of the label claimed content. (FDA. Warning Letters and Test Results for Cannabidiol-Related Products, 2021) In a study of 84 CBD products, only 31% of the products tested were accurately labelled (i.e., within 10% of advertised CBD content) (Bonn-Miller et al. 2017). Another study of CBD products in Mississippi showed that only 2 out of 20 products were within 10% of the advertised CBD content (Gurley et al. 2020). The issue of label accuracy is not unique to the USA. A study in the Netherlands showed that out of 16 CBD oil products tested, only 5 contained CBD within 10% of the label claimed amount (Hazekamp 2018), and a study in Italy found that of 14 CBD oil products tested, only 5 contained CBD consistent within 10% of the labelled content (Pavlovic et al. 2018). In a study from the UK, the researchers reported that 11 of the 29 CBD oil products tested contained CBD within 10% of the advertised amount (Liebling et al. 2022).

For the current study, hemp-derived products (*n* = 80) were purchased at various stores in Central Kentucky and from online retailers from April 2 to May 9, 2021. The products were analyzed for CBD content, and the results were compared to the product label claims. Whereas previous studies have evaluated products available online (Bonn-Miller et al. 2017) or local retailer in a specific state (Gurley et al. 2020), this study investigated both online and local retailers. This study also included the FDA-approved product Epidiolex® as a positive control. Additionally, this study focused solely on oil products as oils were the most prevalent option at time of purchase.

## Methods

### Sample selection

CBD-containing products were acquired via online and brick and mortar retail sources. Of the 80 samples, 44 CBD products were purchased from USA-based online retailers, and the remaining 36 CBD products were purchased from local retailers within Central Kentucky (e.g., CBD shops, head shops, health food markets, and health/wellness stores). The sample set was comprised of 51 different brands (both national and local brands) from 21 online retailers and 9 local (brick and mortar) retailer sources. Epidiolex® (the FDA-approved CBD product) was also obtained (University of Kentucky Investigational Drug Service Pharmacy) to serve as positive control.

Upon purchase, each product was randomly assigned a study identifier to blind researchers to product identification. Products 14 and 15 were lost in shipping and thus not included in the analyses (81 total products including Epidiolex®). Upon receipt, product packaging and seals were inspected to ensure product integrity. The lot numbers were recorded, and the products were stored according to packaging instructions or at room temperature in a dry space if instructions were not provided. All products were tested immediately after opening, and all were tested well before their expiration date.

For product label accuracy, an allowable variance of ± 10% was used, similar to other label accuracy studies (Bonn-Miller et al. 2017), (Pavlovic et al. 2018), (Liebling et al. 2022), with detected CBD concentrations > 110% of labelled value indicating the product was under-labelled (i.e., the product contained more CBD than the label indicated), and detection of < 90% of labelled CBD concentration indicating the product was over-labelled (i.e., the product contained less CBD than label indicated). Products within ± 10% (i.e., 90–110% of labelled value) were categorized as accurately labelled. The observed concentration value was determined by taking the mean of 9 measurements for each sample.

### Reagents and standards

Reference materials were purchased from two different sources for the preparation of calibrator samples and quality control samples. CBD was purchased from the Cayman Chemical (Ann Arbor, MI, USA) for the preparation of calibrator samples and from Dr. Ehrenstorfer (LGC Standards, Manchester, NH, USA) for the preparation of quality control samples. Cannabidiol-d_9_ (CBD-d_9_) was sourced from the Cayman Chemical. Reagents and solvents (LC/MS grade) for use during the extraction and analysis were purchased from Fisher Scientific (Hampton, NH, USA). Extra virgin olive oil, which was used as an analyte-free matrix, was obtained from a local grocery retailer (Kroger, Cincinnati, OH, USA).

### Sample preparation

Prior to analysis, all sample containers were inverted multiple times to ensure contents were thoroughly mixed. Sub-aliquots, 3 replicates of 50 μL, of products were taken and transferred to appropriately labelled containers where internal standard was added at a concentration of 0.020 mg/mL. After mixing, a fixed volume of acetonitrile was added, and the samples were further mixed and then centrifuged (1811 × *g*, 20 min). A 50 μL sub-portion of the supernatant was transferred to an autosampler vial and diluted with solvent and water to form a sample within an appropriate concentration range and composition (nominally 50:50 acetonitrile: water, v:v) for analysis. The samples were capped and briefly vortex mixed prior to analysis by liquid chromatography-tandem mass spectrometry (LC–MS/MS). Samples with analyte concentrations above the calibration range were reanalyzed with dilution (tenfold) prior to internal standard addition.

### Instrumentation

Analysis of samples was carried out via LC–MS/MS using a Thermo Accela 1250 quaternary LC system coupled with a TSQ Vantage mass spectrometer (Waltham, MA, USA). Separations were carried out using a reversed phase (C_8_) Kinetex® analytical column (2.1 − 100 mm, 2.6 μm) purchased from Phenomenex (Torrance, CA, USA). A gradient solvent program was employed using mobile phases of 0.1% formic acid in water (A) and in acetonitrile (B). Briefly, from a starting composition of 50% B, the percentage of organic mobile phase (i.e., B) was increased over 10 min and then an organic flush employed to remove residual matrix components before returning to the solvent starting composition. The solvent flow rate was 500 μL/min, and the total analytical run time was 14.25 min.

The mass spectrometer was equipped with an electrospray ionization (ESI) source operated in positive ion mode using selective reaction monitoring (SRM). Monitored transitions for CBD and its internal standard are listed in Table 1.

**Table 1:** Selective reaction monitoring (SRM) transitions

Analyte	Precursor ion (m/z)	Product ions (m/z)
Cannabidiol	316.2	194.1^a^
		260.2
		123.0
Cannabidiol-d_*9*_	324.2	202.1^a^
		268.2
		123.0

^a^Quantifier ion

### Statistical analysis

For each product sample tested, the measured CBD content was compared to the advertised CBD content on the package label, and the corresponding percentage of label claim was determined. Products were then classified as under-labelled, over-labelled, or accurately labelled, with a 10% tolerance used for classification. Normal-based confidence intervals were then calculated for the proportion of products falling into each category. A secondary analysis partitioned the sample into those purchased online and those purchased locally, and analogous normal-based confidence intervals were calculated for each subset. All calculations were completed using Microsoft Excel.

## Results

A total of 81 CBD products were tested (*n* = 80 unregulated products and the FDA-approved product Epidiolex® [*n* = 1]).

**Fig. 1:**
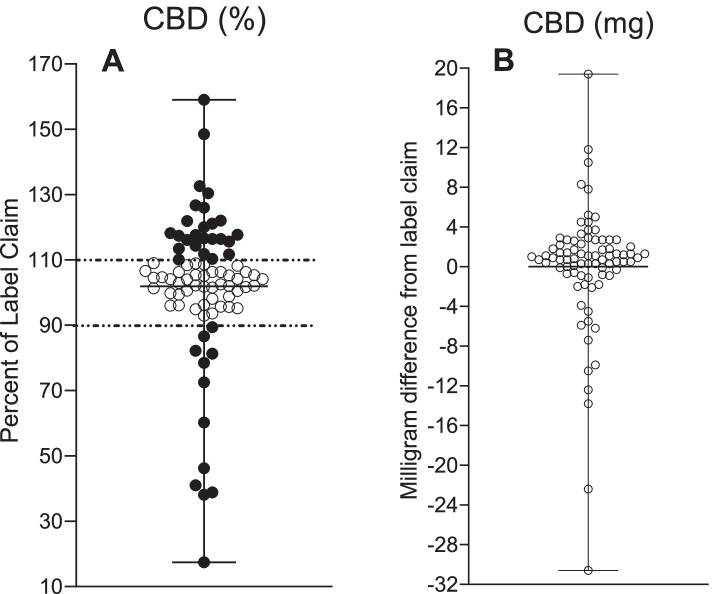
CBD measurements in 80 commercially available CBD oil products and Epidiolex®. **A** The percentage of CBD label claim content with ± 10% tolerance denoting under-labelling (> 110%) and over-labelling (< 90%). **B** Deviation from CBD label claim in milligram

The measured CBD content was compared to the advertised CBD content on the package label, and the percentage of label claim was determined for the CBD content. The CBD content percentage per label claim and milligram deviation per 1 mL of sample from the label claim are shown in Fig. 1. Of the 80 unregulated products tested, 31% [95% *CI*, 21–41%] were under-labelled (*n* = 25), 15% [95% *CI*, 7–23%] were over-labelled (*n* = 12), and 54% [95% *CI*, 43–65%] were accurately labelled (*n* = 44). For the 46% [95% *CI*, 35–57%] of out-of-range samples (*n* = 37), the range of absolute difference was 0.9 to 30.6 mg/mL from the label claim. Epidiolex was within 4% of its labelled concentration (label, 100 mg/mL; analyzed, 96.1 mg/mL) and is represented in the accurately labelled group. Across the unregulated samples tested, the observed CBD concentrations ranged from 2.9 to 61.3 mg/mL, and the values for percent of label claim ranged from 17 (product 13) to 159% (product 79). For under-labelled products (shown in Table 2 including standard error of the mean (SEM)), the average amount of CBD was 121% of label claim, standard deviation of 11%, with a range of 110.1% (product 68) to 159% (product 79). For over-labelled products (shown in Table 3 including SEM), the average percent of label claim was 61%, standard deviation of 24%, with a range of 17% (product 13) to 89% (product 76).

**Table 2:** List of samples containing at least 10% more CBD than label claim (i.e., under-labelled samples)

**Sample identifier**	**Source**	**Label claim mg CBD/mL**	**Observed mg CBD/mL ± SEM (mg/mL)**	**Difference mg CBD/mL**	**Percent of label claim**
79	Online	20.0	31.8 (± 0.5)	11.8	159
12	Online	40.0	59.4 (± 0.8)	19.4	148
40	Local	10.0	13.3 (± 0.2)	3.3	133
2	Local	17.0	22.2 (± 0.3)	5.2	130
59	Local	10.0	12.7 (± 0.2)	2.7	127
58	Local	30.0	37.8 (± 0.7)	7.8	126
3	Online	17.0	20.7 (± 0.2)	3.7	122
1	Local	17.0	20.7 (± 0.2)	3.7	122
77	Online	50.0	60.5 (± 1.2)	10.5	121
47	Local	25.0	30.0 (± 0.4)	5.0	120
43	Local	25.0	29.5 (± 0.4)	4.5	118
33	Local	10.3	12.2 (± 0.1)	1.8	118
39	Local	10.0	11.8 (± 0.1)	1.8	118
38	Local	10.0	11.7 (± 0.1)	1.7	117
4	Online	50.0	58.3 (± 1.1)	8.3	117
22	Online	16.7	19.4 (± 0.4)	2.7	116
26	Local	16.7	19.4 (± 0.3)	2.7	116
37	Local	8.3	9.7 (± 0.1)	1.3	116
81	Online	16.7	19.3 (± 0.3)	2.6	116
36	Local	8.3	9.5 (± 0.1)	1.2	114
66	Online	16.7	18.9 (± 0.3)	2.2	113
67	Online	16.7	18.6 (± 0.3)	2.0	112
42	Local	25.0	27.9 (± 0.4)	2.9	112
11	Online	8.4	9.3 (± 0.1)	0.9	110
68	Online	16.7	18.3 (± 0.2)	1.7	110
Mean		19.7	24.1	4.5	121
Standard deviation		11.8	15.3	4.2	11

**Table 3:** List of samples containing at least 10% less CBD than label claim (i.e., over-labelled samples)

Sample identifier	Source	Label claim mg CBD/mL	Observed mg CBD/mL ± SEM (mg/mL)	Difference mg CBD/mL	Percent of label claim
76	Online	16.7	14.9 (± 0.2)	− 1.8	89
30	Local	33.3	28.9 (± 0.2)	− 4.5	87
5	Online	33.3	27.4 (± 0.2)	− 5.9	82
7	Online	33.3	27.1 (± 0.3)	− 6.2	81
29	Local	34.5	27.1 (± 0.4)	− 7.4	79
80	Online	20.0	14.5 (± 0.7)	− 5.5	73
31	Local	25.0	15.1 (± 0.2)	− 9.9	60
28	Local	41.7	19.2 (± 0.2)	− 22.4	46
48	Local	17.9	7.3 (± 0.1)	− 10.5	41
24	Online	50.0	19.4 (± 0.4)	− 30.6	39
45	Local	20.0	7.6 (± 0.1)	− 12.4	38
13	Online	16.7	2.9 (± < 0.1)	− 13.8	17
Mean		28.5	17.6	− 10.9	61
Standard deviation		10.8	8.8	8.2	24

Of the 80 nonregulated CBD oil products (excluding Epidiolex®), 44 products were acquired through online retailers and 36 products purchased at local retailers in Central Kentucky. Of the 44 online products, 25% [95% *CI*, 12–38%] (*n* = 11) were under-labelled, 14% [95% *CI*, 3–24%] (*n* = 6) were over-labelled, and 61% [95% *CI*, 47–76%] (*n* = 27) were accurately labelled for CBD content. Of the 36 locally purchased products, 39% [95% *CI*, 23–55%] (*n* = 14) were under-labelled, 17% [95% *CI*, 5–29%] (*n* = 6) were over-labelled, and 44% [95% *CI*, 28–61%] (*n* = 16) were accurately labelled.

## Discussion

Recent studies of CBD products have led to quality concerns regarding the accuracy of product labelling especially with regard to CBD content (Gurley et al. 2020), (Bonn-Miller et al. 2017), (Hazekamp 2018), (Pavlovic et al. 2018), (Liebling et al. 2022). For this study, 80 unregulated CBD oil products were purchased from online retailers and local retailers in Central Kentucky. The products purchased for the study represented the range of CBD product manufacturers from local small businesses to companies with nationwide distribution, and Epidiolex® was included as a positive control. Of the products tested, 54% were found to have CBD concentrations consistent with the advertised amount on the label while 31% were found to contain more than 110% of the label claim, and 15% were found to contain less than 90% of the label claim amount of CBD. The results of this study are consistent with the findings of these previous studies.

Since December 2018, hemp-derived CBD products have inundated the US market in a variety of forms including ingestible oils, gummies, beverages, topical creams, and inhalation liquids (i.e., vape pens), with sublingual oils being the most common (Corroon and Phillips 2018). During this time, the regulatory status of CBD oils has been vague and imprecise. Consumers have increasingly explored and used CBD oils for the purported benefits primarily as a specific therapy for medical conditions and secondarily for general health and well-being (Corroon and Phillips 2018). Corroon and Phillips reported that consumers are taking CBD products to treat multiple medical conditions, with an average of 2.67 medical conditions per consumer. However, there is a disconnect between the products that consumers are actually taking (i.e., the unregulated CBD products analyzed here) and the CBD products that are being tested in clinical trials (e.g., Epidiolex®,other pharmaceutical grade/regulated products) (Gurley et al. 2020). In addition, although Epidiolex® is currently FDA approved for the treatment of three seizure conditions, CBD has not been FDA approved to treat other conditions. Despite its popularity for the treatment of pain, anxiety, insomnia, and other conditions, there is not substantial scientific evidence to support its use for these conditions (due to little to no controlled data or data that suggest little to no efficacy) (Cannabidiol: critical review report. Expert Committee on Drug Dependence 2018; Britch et al. 2021). Despite this lack of empirical evidence, consumers are learning about CBD and often determining their own treatment plans from anecdotal evidence acquired from Internet research, family members, or friends (Corroon and Phillips 2018). As consumers are taking CBD products without medical guidance, it is imperative that, at a minimum, product labels convey clear and accurate information on CBD content to best allow the consumer to be accurately informed about the doses that they are taking. The inaccuracy of labelling means that vulnerable consumers will not receive the expected dose of CBD leading to concerns with respect to efficacy, side effects, and consumer safety. With the range of CBD concentrations available to consumers, 7.5 to 60 mg/mL in this study, even small percentages of label inaccuracy could result in significant variation of CBD dosage from the intended dose, especially considering the potential for dosing multiple times per day.

The bioavailability of CBD has been estimated to be 6% due to extensive first pass metabolism (Cannabidiol: critical review report. Expert Committee on Drug Dependence, 2018). CBD metabolism occurs in the liver through the actions of cytochrome P450 isozymes (Jiang et al. 2011; Samanta 2019). More specifically, the primary metabolites of CBD, 7-hydroxy-CBD, and 6-hydroxy-CBD have been shown to be mediated by CYP2C19 and CYP3A4 (Jiang et al. 2011). In vitro, Bansal et al. reported time-dependent inhibition of CYP1A2, CYP2C19, and CYP3A, demonstrated by a decrease in activity of 83%, 75%, and 85%, respectively (Bansal et al. 2020). Clinical studies of epilepsy using Epidiolex® have demonstrated potential inhibition or induction of CYP2C19, CYP3A4, CYP2C8, CYP2C9, CYP1A2, CYP2B6, UGT1A9, and UGT2B7 (Samanta 2019), (Epidiolex: prescribing informtion. 2018). The risk of dose-dependent drug-drug interaction (when high doses of CBD are taken in combination with other medications and/or dietary supplements) emphasizes the need for accuracy in labelling to better assist the consumer/practitioners to determine appropriate dosing.

High doses of CBD can also lead to hepatocellular injury/toxicity (Epidiolex: prescribing informtion 2018). In a study of acute and subacute toxicity, CBD dose dependently increases both alanine aminotransferase (ALT) and aspartate aminotransferase (AST) along with an increase of liver-to-body weight ratios and increased total bilirubin (Ewing et al. 2019). In some clinical trials, elevated liver aminotransferase enzyme levels were > 3 times the upper limit of the normal range and led to patient withdrawal (Devinsky et al. 2017; Leehey et al. 2020). Additionally, Ewing et al. showed differential regulation of more than 50 gene markers related to hepatotoxicity after administration of CBD (Ewing et al. 2019). Elevation of markers of liver injury after administration of CBD has been shown to occur in a dose-dependent manner. Label accuracy is important for consumers since hepatocellular injury is dose dependent especially with consideration of concomitant drug administration.

Limitations of the presented data include the following: (1) the analysis of only hemp-derived oil products to the exclusion of other product types such as gummies, topicals, and vapes — at the time of purchase, oils were the most prevalent option available; (2) reporting only CBD concentrations but no other cannabinoid concentrations; and (3) not implementing a formal sampling protocol (i.e., a priori distribution of online, national and local brands; although all are represented in the current data).

## Conclusions

The results of this study add to the evidence from several countries demonstrating that CBD content in over-the-counter CBD oil products is often inconsistent with the label claims. Inaccurate labelling has the potential to present safety risks to the consumer. As most consumers are using CBD products as therapeutic treatments for some types of medical condition, the dosing is important when considering the potential for CBD accumulation, elevation of liver enzymes, and drug-drug interactions. The findings reported here emphasize the continued need for clear and consistent regulation from federal and state agencies to ensure label accuracy of CBD products and subsequent enforcement. These results also indicate the need for continued development of good manufacturing practices and testing standards. As consumers are taking CBD products for an ever-increasing range of conditions, independent of medical guidance, the accuracy of content labelling is important for the safety of the consumer.

## Data Availability

The dataset supporting the conclusions of this article is included within the article. The raw data can be made available for review upon request.

## Abbreviations

CBD: Cannabidiol
Δ^9^-THC: Δ^9^-Tetrahydrocannabinol
CI: Confidence interval
LC–MS/MS: Liquid chromatography-tandem mass spectrometry
FDA: United States Food and Drug Administration

## References

[CR1] Agricultural Improvement Act, (2018). https://www.congress.gov/115/plaws/publ334/PLAW-115publ334.pdf

[CR2] Bansal S, Maharao N, Paine MF, Unadkat JD (2020). Predicting the potential for cannabinoids to precipitate pharmacokinetic drug interactions via reversible inhibition or inactivation of major cytochromes P450. Drug Metab Dispos.

[CR3] Bonn-Miller MO, Loflin MJE, Thomas BF, Marcu JP, Hyke T, Vandrey R (2017). Labeling accuracy of cannabidiol extracts sold online. JAMA.

[CR4] Britch SC, Babalonis S, Walsh SL (2021). Cannabidiol: pharmacology and therapeutic targets. Psychopharmacology.

[CR5] Cannabidiol: critical review report. Expert Committee on Drug Dependence, 40th Meeting.: World Health Organization; 2018. https://www.who.int/medicines/access/controlled-substances/CannabidiolCriticalReview.pdf

[CR6] Corroon J, Kight R (2018). Regulatory status of cannabidiol in the United States: a perspective. Cannabis Cannabinoid Res.

[CR7] Corroon J, Phillips JA (2018). A cross-sectional study of cannabidiol users. Cannabis Cannabinoid Res.

[CR8] Devinsky O, Cross JH, Laux L, Marsh E, Miller I, Nabbout R (2017). Trial of cannabidiol for drug-resistant seizures in the Dravet syndrome. N Engl J Med.

[CR9] Epidiolex: prescribing informtion. In: Administration UFaD, editor. 2018. https://www.accessdata.fda.gov/drugsatfda_docs/label/2018/210365lbl.pdf

[CR10] Ewing LE, Skinner CM, Quick CM, Kennon-McGill S, McGill MR, Walker LA (2019). Hepatotoxicity of a cannabidiol-rich cannabis extract in the mouse model. Molecules.

[CR11] FDA approves new indication for drug containing an active ingredient derived from cannabis to treat seizures in rare genetic disease [press release]. July 31, 2020. https://www.fda.gov/news-events/press-announcements/fda-approves-new-indication-drug-containing-active-ingredient-derived-cannabis-treat-seizures-rare

[CR12] FDA. Warning Letters and test results for cannabidiol-related products [Internet]. FDA; [updated August 5, 2021. Available from: https://www.fda.gov/news-events/public-health-focus/warning-letters-and-test-results-cannabidiol-related-products.

[CR13] Gurley BJ, Murphy TP, Gul W, Walker LA, ElSohly M (2020). Content versus label claims in cannabidiol (CBD)-containing products obtained from commercial outlets in the state of Mississippi. J Dietary Suppl.

[CR14] Hazekamp A (2018). The trouble with CBD oil. Med Cannabis Cannabinoids.

[CR15] Jiang R, Yamaori S, Takeda S, Yamamoto I, Watanabe K (2011). Identification of cytochrome P450 enzymes responsible for metabolism of cannabidiol by human liver microsomes. Life Sci.

[CR16] Leehey MA, Liu Y, Hart F, Epstein C, Cook M, Sillau S (2020). Safety and tolerability of cannabidiol in Parkinson disease: an open label, dose-escalation study. Cannabis Cannabinoid Res.

[CR17] Liebling JPC, Nicholas J, Gibbs BW, Yates ASY, O’Sullivan SE. An analysis of over-the-counter cannabidiol products in the United Kingdom. Cannabis Cannabinoid Res. 2022;7(2):207–13. 10.1089/can.2019.0078. 10.1089/can.2019.0078 PMC9070743 33998849

[CR18] Pavlovic R, Nenna G, Calvi L, Panseri S, Borgonovo G, Giupponi L (2018). Quality traits of “cannabidiol oils”: cannabinoids content, terpene fingerprint and oxidation stability of European commercially available preparations. Molecules.

[CR19] Samanta D (2019). Cannabidiol: a review of clinical efficacy and safety in epilepsy. Pediatr Neurol.

[CR20] Scientific data and information about products containing cannabis or cannabis-derived compounds; Public Hearing [press release]. May 31, 2019. https://www.fda.gov/news-events/fda-meetings-conferences-and-workshops/scientific-data-and-information-about-products-containing-cannabis-or-cannabis-derived-compounds 10.1177/1535759719878716 PMC6891184 31640419

[CR21] Wagoner KG, Lazard AJ, Romero-Sandoval EA, Reboussin BA (2021). Health claims about cannabidiol products: a retrospective analysis of U.S. Food and Drug Administration warning letters from 2015 to 2019. Cannabis Cannabinoid Res..

